# A Pragmatic Approach for Rapid, Non-Destructive Assessment of Defect Types in Laser Powder Bed Fusion Based on Melt Pool Monitoring Data

**DOI:** 10.3390/ma17133287

**Published:** 2024-07-03

**Authors:** Anna Engelhardt, Thomas Wegener, Thomas Niendorf

**Affiliations:** Institute of Materials Engineering, Metallic Materials, University of Kassel, Mönchebergstraße 3, 34125 Kassel, Germany; t.wegener@uni-kassel.de (T.W.); niendorf@uni-kassel.de (T.N.)

**Keywords:** additive manufacturing, selective laser melting, process monitoring, AlSi10Mg, MPM

## Abstract

Process monitoring systems, e.g., systems based on photodiodes, could be used in laser-based powder bed fusion (PBF-LB/M) to measure various process parameters and process signatures to eventually allow for a local, detailed analysis of the produced parts. Here, simple statements only concerning the occurrence of defects in parts are sufficient in many cases, especially with respect to industrial application. Therefore, a pragmatic approach to rapidly infer the occurrence of defects and their types based on in situ data obtained by commercially available process monitoring systems is introduced. In this approach, a color distribution in form of a histogram is determined for each produced part using layer-wise screenshots of the visualized data provided by the monitoring software. Assessment of the histograms of AlSi10Mg samples, which were processed with different parameter combinations, revealed characteristics depending on the prevailing defect types. These characteristics enable the prediction of the occurring defect types without the necessity to apply conventional downstream testing methods, and thus, a straightforward separation of parts with good quality from defective components. Since the approach presented uses the data visualization of the monitoring software, it can be used even when direct access to the raw data is not provided by the machine manufacturer.

## 1. Introduction

Powder bed-based additive manufacturing (AM) techniques have become one of the most promising candidates in the manufacturing of light-weight components with complex geometry during recent years [1]. Laser-based powder bed fusion of metals (PBF-LB/M), also referred to as selective laser melting (SLM), represents the most commonly used powder bed-based AM process [2,3]. The PBF-LB/M process is based on the consecutive consolidation of metallic powders by a fiber laser, and the selective and repetitive fusing of thin layers on top of each other, enabling a tool-free near-net shape production of complex freeform parts directly from a computer-aided design (CAD) model [4,5,6]. A major challenge in PBF-LB/M continues to be the assurance of process robustness, stability, repeatability and, eventually, a high part quality [7,8]. This especially holds true for industrial sectors being characterized by stringent requirements and certification constraints like medical and aerospace. Furthermore, the avoidance of common defect types like unmelted regions (so-called lack of fusion (LoF) defects), cracks, or keyhole porosity [9] is important in many applications, since defects like LoF or keyhole porosity negatively affect the mechanical properties of parts [10]. So, generally, an extensive quality inspection is necessary in order to ensure high quality, e.g., in form of low porosity, and thus superior structural integrity (SI) of the components [9]. Often, this quality inspection is done after the manufacturing using conventional destructive or non-destructive testing methods, e.g., metallography, computed tomography ((µ-)CT), or tensile testing [11]. Since this downstream quality inspection can be a time- and cost-consuming step, different approaches for quality inspection during the manufacturing process, referred to as process monitoring, have been proposed [7]. During process monitoring, specific key process parameters and process signatures (“dynamic characteristics of the powder heating, melting, and solidification processes as they occur during the build” [12]), which correlate with the properties of the manufactured parts, e.g., density, dimensional accuracy, surface finish, and mechanical properties, are measured during the process with in situ sensing devices [7,8]. Based on the measured data, process-induced defects and anomalies can be detected and localized using in-process data analytics and statistical monitoring techniques. Therefore, the quality and stability of the process can be determined during manufacturing [8,13], a non-destructive quality inspection becomes possible, and time- and cost-consuming conventional testing methods can be avoided. Moreover, failed parts can be detected and removed during manufacturing, eventually reducing production time [13]. In combination with the knowledge on the relationship of the process parameters and the in situ measured key process parameters and process signatures, a real-time closed-loop control, e.g., for repairing actions, could also be realized [7,8]. Besides, the whole manufacturing process is documented by the process monitoring system [13]. In addition, the recorded data can help to gain insights in the process itself, e.g., the interaction between the beam and the material, the cooling history, or the presence of by-products [14].

Process monitoring is a wide field with different approaches to monitor a wide range of key process parameters, e.g., laser power and process signatures like powder bed surface and part distortion [8,15]. Nevertheless, the most common process signatures monitored are the spatial and temporal temperature conditions within or close to the laser–material interaction zone [15]. The temperature field strongly correlates with the part quality, since it has a direct impact on the resulting microstructure, density, and mechanical properties of the part [7]. According to Zeng et al. [16], a homogenous temperature field during manufacturing results in better part quality (microstructure, mechanical properties, dimensional accuracy, surface finish). In most applications, a contactless measurement with pyrometers in form of digital cameras or photodiodes is used to monitor the temperature field within or close to the laser–material interaction zone [7,15]. Some of the approaches considered are offered in combination with conventional PBF-LB/M systems, e.g., the Melt Pool Monitoring (MPM) from SLM Solutions Group AG (Lübeck, Germany). As detailed in [13,17], the MPM measures the thermal emission during the build job using two photodiodes with a frequency of up to 100 kHz. The MPM was developed to provide a detailed representation of the thermal emissions, so that the entire manufacturing process can be documented and part quality can be assured. Moreover, the MPM was designed to generate further process understanding and enable a quasi closed-loop control function, which eventually should allow for an adjustment of the laser power in about 60 μs. However, the control function is only available for development purposes at the moment [17].

Besides the MPM, other melt pool monitoring systems offered in combination with conventional PBF-LB/M systems exist, e.g., QM Meltpool 3D from Concept Laser GmbH (Lichtenfels, Germany), EOSTATE MeltPool from EOS GmbH (Krailling, Germany), and MeltVIEW from Renishaw plc (Wotton-under-Edge, UK). These systems have similar representations of the measured values, and their potential for defect detection was investigated in different studies. Since the present work solely focusses on the MPM of SLM Solutions AG, the reader is referred to the open literature, e.g., [18,19,20], for more information about the other mentioned systems.

Looking at the state of the art with respect to the MPM of SLM Solutions AG, only a few studies can be found in open literature. Alberts et al. [13] investigated the influence of different processing parameter combinations on the experimentally determined MPM data during the PBF-LB/M of Inconel 718 samples and reported a correlation between the variations of processing parameters like layer thickness, laser power, scan velocity, or hatch distance, and the measured thermal emission as well as the part density [13]. In the study of Artzt et al. [21], MPM data obtained during the manufacturing of Ti-6Al-4V were analyzed considering surface-near areas of samples produced with different scan patterns. A correlation between the quotient ADC2/ADC1, the surface roughness, and residual stress was pointed out by the authors. Yadav et al. [22] developed a machine learning (ML) based approach to identify layers with hotspots using MPM data. In a second study, Yadav et al. [23] combined another ML approach with a statistical method to identify hotspots in MPM data at global and local scales, comparing the obtained results with results of a layer control system as well as CT scans. However, in these studies ([13,21,22,23]) MPM raw data were used that are not available for commercial users. Thus, the described approaches are not applicable for most users and the use of the MPM as non-destructive quality inspection tool is limited so far.

In the study of Mohr et al. [15], the PBF-LB/M of an AISI 316L stainless steel sample with artificial defects and areas processed with different parameter combinations was assessed using different monitoring systems, including the MPM among others. Although a reasonable correlation of defects with the results of downstream µ-CT investigations was reported, a more detailed investigation was not possible due to the missing option of exporting the MPM raw data and missing details about the equipment, the measurement principle, and the displayed signal [15].

As pointed out by the studies presented, MPM data have the potential to substantiate information on part quality. As already detailed, the use of the MPM as non-destructive quality inspection tool is limited for commercial users at the moment, as was underlined by the study of Mohr et al. [15]. These limitations result from different aspects. One obstacle in the use of MPM data is the missing option of exporting the data. As a result, the use and investigation of the data is hindered so that only a few studies deal with MPM data.Moreover, open questions concerning the correlations of data and part properties still exist. Therefore, even a separation of parts with good quality from defective components based on the MPM data is not possible at the moment. With respect to industrial applications, such a separation would be a sufficient and beneficial non-destructive quality inspection in many cases.

In order to close the prevailing research gaps, an alternative approach that is independent of the accessibility of raw data is needed. Such an approach would enable an open, versatile analysis of the data for every user, so that further research can be conducted to answer open questions and to gain further insights into the correlation to quality features like the occurring part defects. Based on those research results, the use of the MPM as non-destructive quality inspection tool might become possible. Therefore, an approach is introduced in the present study that is based on layer-wise screenshots of the data representation in the MPM software, and that enables the use of MPM data despite the missing export option. In the approach detailed, screenshots were analyzed concerning the displayed colors to obtain histograms. The approach was applied to MPM data gained during a previous parameter study on PBF-LB/M of AlSi10Mg [24]. Characteristics in the resulting histograms could be related to observed part defects (referred to as defect types in the remainder of this manuscript). These characteristics allow the prediction of defect types for new and unknown samples, so that a separation of parts with good quality from defective components without conventional, time-consuming downstream testing methods like metallography or µ-CT is enabled. As a result, the MPM can be used as a fast and non-destructive quality inspection tool.

The presented approach does not require the use of ML or Artificial Intelligence (AI) so that it is easy to use. However, the approach likely enables the use of such tools in future work as it provides access to the MPM data. Using ML or AI techniques, further improvement of the approach presented, e.g., by a local analysis of defect formation, which is not part of the approach presented at this point, is expected to be realized.

## 2. Method and Materials

### 2.1. Sample Manufacturing and Investigation of Porosity

To investigate possible relationships of the MPM data and properties of the resulting parts like defects, a huge data base is necessary. This data base must include samples with different defect types caused by different process parameter combinations. A significant number of different process parameter combinations was investigated for PBF-LB/M processed AlSi10Mg in a previous study [24]. Therefore, the corresponding MPM data were chosen as data base of the present work. In the following, a short summary of the most important aspects concerning the data base is given. For further details on sample manufacturing and the subsequent investigation of defect types and porosity, the reader is referred to the previous study [24].

The data base contains 144 samples processed using 112 different parameter combinations. The used parameter combinations follow a space-filling experimental design (Latin hypercube design) that was combined with several repetitions, and some manually chosen parameter combinations. Four different layer thicknesses $s\in \left\{30,\;45,\;60,\;90\right\}$ µm were considered, and for each s the influence of laser power P, scan speed $v_{s}$, and hatching distance h was investigated in wide ranges (P=[200, 400] W for s={30, 45} µm, P=[250, 400] W for s=60 µm and P=[300, 400] W for s=90 µm, $v_{s}=[350,\;1750]$ mm s^−1^, h=[0.1, 0.4] mm. For the exact parameter combinations, the reader is referred to the data publication of the previous study [25].). The samples were divided into six groups with 24 samples each. Each group was built as one build job using a SLM 280^HL^ PBF-LB/M system from SLM Solutions Group AG (Lübeck, Germany) equipped with a substrate plate made of an aluminum alloy. The samples were manufactured in form of cubes with an edge length of 10 mm built on 5 mm high supports (type: block supports) using AlSi10Mg powder produced by TLS Technik GmbH & Co Spezialpulver KG (Bitterfeld-Wolfen, Germany) with a particle size ranging from 20 µm to 63 µm. The production of the samples took place under an argon atmosphere, with an oxygen level below 0.2%, at a build plate temperature of 100 °C using a 400 W fiber laser characterized by a Gaussian beam profile (beam diameter: 0.08 mm). A line scanning strategy with a rotation of the scanning direction by 90° in each layer and contour passes (P=400 W and $v_{s}=1170$ mm s^−1^) were applied. During the manufacturing process, the MPM (Version 4.4) was employed to record data with a data rate of 10 µs. As already mentioned in Section 1, the MPM measures the thermal emission during the build job using two photodiodes [13,17]. The photodiodes have different photosensitivity in the near-infrared range (wavelength ranges up to 2500 nm [26]), and their measured values are referred to as ADC1 and ADC2 (the signals of the photodiodes are sent to an analog digital converter (ADC)). For a schematic detailing the mounting of the MPM in the SLM system, the reader is referred to [13,17]. Unfortunately, no further information from the system manufacturer on the photodiodes or the mounting of the system is provided. However, information can possibly be transferred from the in situ process monitoring system used in Renishaw machines since this system uses the same approach as the MPM (two photodiodes with different photosensitivity). Respective information is detailed in [27]. In this system, the different photosensitivities of the photodiodes are used to obtain information concerning different emissions: the plasma emission (photodiode 1) and the infrared emission (photodiode 2) [27].

After processing, samples were embedded in resin and ground down to 5 µm grit size parallel to the building direction (BD). All samples were examined using an AxioPlan optical microscope from Carl Zeiss Microscopy GmbH (Jena, Germany) at 50 × magnification with a resolution of 2.056 µm/Pixel. A series of images was taken from each sample so that the sample could be imaged as a whole. The single images were stitched using the Adobe Photoshop CS2 (version 9.0, Adobe Systems Incorporated, San Jose, CA, USA) function “Photomerge”. As last step, the complete image of every sample was evaluated with respect to porosity and the occurring defect type was determined.

Different defect types could be observed in the complete images. Considering a constant s, the occurrence of defect types could be related to the energy density

(1) $$E=\frac{P}{v_{s}\cdot h\cdot s}$$

Parameter combinations characterized by a low E lead to LoF or, if a wide h is used, to so-called “stripe pores”. Both defects occur between adjacent scan tracks due to a non-adequate overlap but are characterized by different appearances. LoF defects are large pores with irregular shapes occurring randomly. Stripe pores are long, straight cavities ranging from the bottom of the sample to its top and occur in defined distances. Nearly fully dense material could be obtained with parameter combinations related to an optimal E range depending on the used s. Using parameter combinations characterized by a high E, porosity that is present in form of small circular pores occurs. This defect type is referred to as keyhole porosity in the following. Here, it has to be noted that this is a simplified consideration, and the combination of the single process parameters always has to be considered [24] since P and $v_{s}$ define the melt pool geometry and h the position of the melt pools in relation to each other. melt pool geometry and position are important factors in the defect evolution [28,29].

### 2.2. Analysis of MPM Data

In the MPM software, the data of the two photodiodes called ADC1 and ADC2 and two ratio combinations (ADC1/ADC2 and ADC2/ADC1) of those results can be displayed. Data are displayed in form of an x–y diagram using a color scale consisting of 63 different colors. Each color represents a range of measured values. Considering the used color scale, the color blue stands for a small and the color red for a high measured value, respectively. A detailed list of colors belonging to color classes can be found in the Supplementary Materials. Since no labeling is given for the color scale in the MPM software, the direct interpretation of the data shown is difficult. However, due to the MPM results given as thermal emission in counts in [13] and the operating mode of photodiodes detailed in [7], the displayed signal is most probably related to the intensity of the light reaching the sensitive part of the photodiode and, thus, to the temperature of the probed area [7]. With this assumption, a higher MPM value implies a higher temperature in the area of the melt pool. The color scale of the data shown is adjusted to every layer automatically. To show the data with a constant color scale and to enable a direct comparison of the results of different layers, the option “Fixed color scale” can be activated. In this case, the color scale of the actual layer is set and used to display all results.

The data displayed in the MPM software can be viewed at different magnification levels. Moreover, one of the three modes “hottest value”, “average value”, and “coldest value” can be selected. Unfortunately, a clear definition of the three modes was not provided by the manufacturer, but most probably these three modes are defined due to the limitation by the pixels of computer screens. Imposed by the high number of measured values, not every value can be displayed in the MPM software by one pixel, especially at low magnification levels. Therefore, measured values of adjacent spots must be reduced to a representative value that can be displayed by one pixel. Here, it is assumed that the three modes are used to point at the calculation of the representative values by using the hottest, average, or coldest value as representative value.

**Figure 1:**
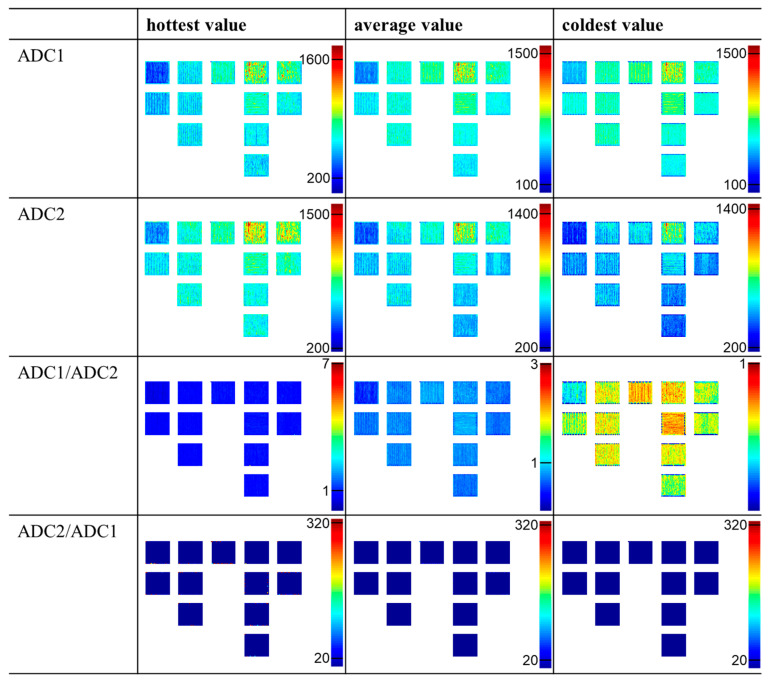
Different representations of data in the MPM software, exemplary taken from the middle of the sample height from a build job of PBF-LB/M processed AlSi10Mg using s=30 µm. The results of the two photodiodes (ADC1 and ADC2) can be displayed separately or in relation to each other. All shown representations have their individual color scale, which was adjusted automatically by the MPM. The color scale has no labeling in the MPM software, but following the results described in [13], the color scale shows the thermal emission in counts.

An overview of the potential representations of the different data (ADC1, ADC2, ADC1/ADC2, and ADC2/ADC1) and modes (“hottest value”, “average value”, and “coldest value”) is exemplary shown in Figure 1 for a representative layer of 12 PBF-LB/M processed AlSi10Mg samples. The layer was always taken from the middle of the sample height. As can be seen, the signals of ADC1 and ADC2 generally result in similar representations, although they vary in details. The measured signals of ADC2 are slightly lower than the signals of ADC1, confirming the different photosensitivity of both photodiodes. For the layer displayed (especially the representations of the “hottest value” mode), data plotted are characterized by pronounced similarity, while the representations of the “coldest value” mode vary significantly. As a result, the ratio of both values ADC1/ADC2 shows a nearly uniform signal for the “hottest value” mode and more details in case of the “coldest value” mode. Nevertheless, in all three modes, several values of ADC1/ADC2 are close to 1, indicating that both values are equal. Therefore, it is questionable if the ratio of both values has a great significance or if important details might get lost by analysis of this value with respect to the aims of the present study. The other ratio ADC2/ADC1 results in a uniform signal independent of the mode. Although a few values vary from this signal, this ratio contains less information and is thus not suitable for further analysis in the present work. Therefore, in order to gain first insights into the correlation of MPM data and defects, only results of ADC1, displayed in the “hottest value” mode, were used in the present study.

**Figure 2:**
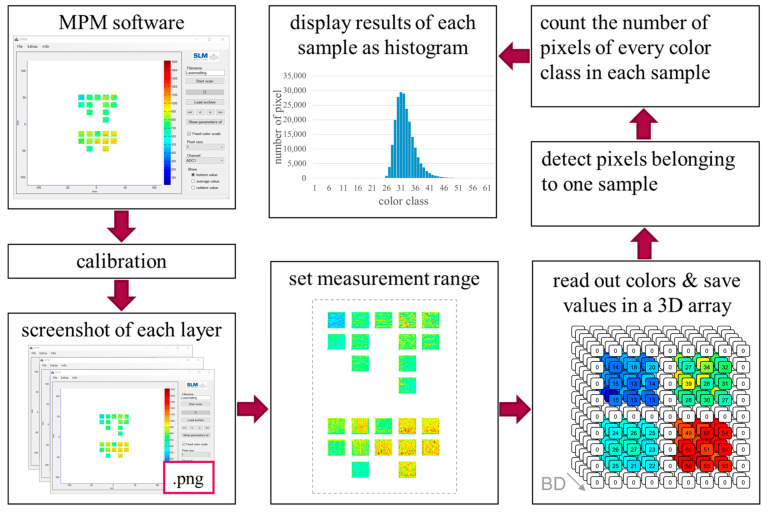
Workflow for the analysis of MPM data. The values in the 3D array are not based on real measurements, but illustrate the concept of the three-dimensional array.

The workflow used to analyze the MPM data is shown in Figure 2 and explained in detail in the following. As first step, the color scale was fixed using a randomly selected layer. As a consequence, the 63 colors of the color scale represent measured values in a range of 0 to 1500 counts (last value shown in the scale), with one color representing a value range of approximately 24 counts. After this initial step, a screenshot of every layer was taken and saved in.png format. This step was necessary to compensate the missing option to export data in the used software version. To be able to further analyze these screenshots, it is important that each sample is displayed as a continuous area. In the present study, the considered samples were displayed as almost squared rectangles with an edge length between 24 Pixel and 32 Pixel. Eventually, a resolution between 0.31 mm/Pixel and 0.42 mm/Pixel was considered. The differences in the edge length and the resolution are due to the magnification function of the MPM. Here, a rectangle enclosing the area that should be enlarged is drawn by the user. Since there is no grid or other supporting options, a repeatable magnification can only be achieved with very high effort and a high amount of trial and error. The used resolutions were in the range of the highest mm/Pixel resolution that can be applied. This maximum resolution is defined by the MPM software. A standard view exists in the MPM software that marks the lowest useable magnification and, with it, the highest usable resolution. Cubic samples with an edge length of 10 mm are displayed in the standard view as almost-squared rectangles with an edge length between 23 Pixel and 26 Pixel. This results in a resolution of 0.43 mm/Pixel. Moreover, the required representation of the data in form of a continuous area limits the applicable magnification level used in the MPM software and, with it, the lowest usable resolution (given in mm/Pixel). However, with approaches based on, e.g., clustering methods, the use of representations with higher magnification levels will become possible in the future.

To analyze the data represented by the screenshots, an algorithm was developed. As first step of this algorithm, a measurement range was set containing the entire area of the samples. Within this area of interest, the color of every pixel was read, converted to a so-called color class, defined by numbers from 1 (low values/blue) to 63 (high values/red) or the value 0 in case of a white pixel, and saved into a three-dimensional array (3D array). Considering the 3D array, all pixels belonging to one sample were identified by the algorithm. Therefore, adjacent pixels with a color different than white were marked as related pixels. As a next step, the number of pixels of each color class in one sample was calculated. Since each color class represents a range of measured values, the overall fraction of the corresponding range of measured values is actually determined in this step. These results were plotted as number of pixels vs. the color classes in histograms. So, the histograms are actually a simplified illustration of the distribution of the measured values.

This simplification results from different data reduction steps related to the use of screenshots. The basic idea of all these data reduction steps is to summarize values into groups or classes. The first data reduction step is done by the MPM software itself with the use of the “hottest value”, “average value”, or “coldest value” mode. As already detailed, these modes probably combine a group of measured values to one value. This data reduction step is strongly related to the chosen magnification that defines the number of pixels used to display one sample. With the use of a low magnification, the number of pixels decreases and the data reduction is more significant as the total number of measured values that were reduced to one value becomes higher. The resulting values are displayed using 63 colors. Every color represents a range of measured values, thus generating an additional data reduction step. As a result, the number of evaluable values is clearly reduced. However, as pointed out by Grasso et al. [8], data reduction techniques like dimensionality reduction will play an important role in the development of process monitoring tools, as big data streams at high frequency (in case of the MPM, up to 100 kHz [13,17]) are generated during the manufacturing process. An example of data reduction using MPM data can be found in the study of Artzt et al. [21]. There, data reduction was realized by considering each fifth measurement value only [21].

## 3. Results and Discussion

### 3.1. Characteristics in Histograms of Complete Samples

**Figure 3:**
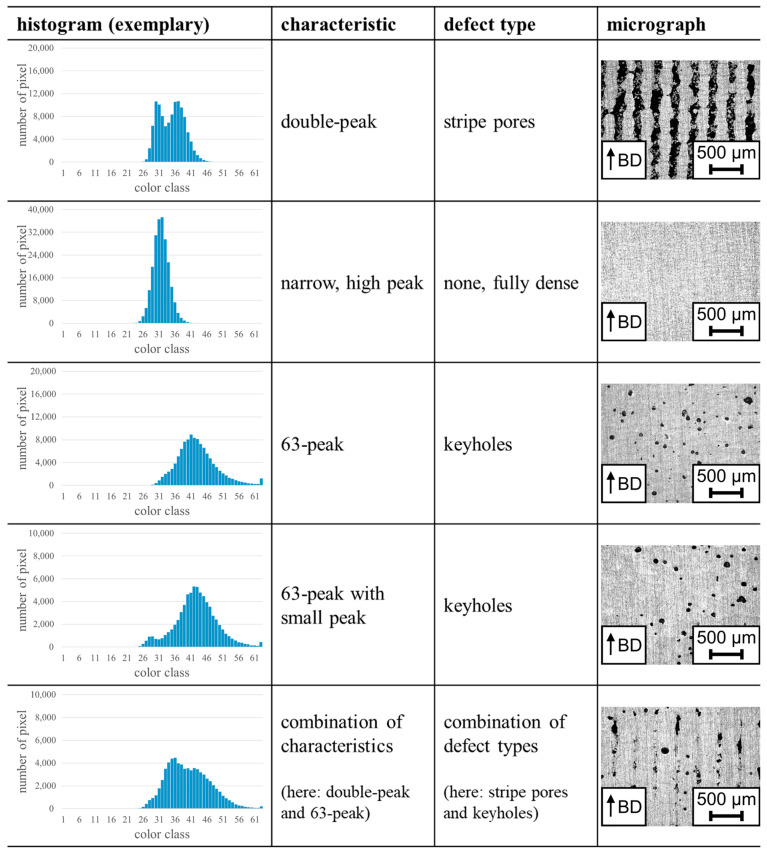
Representative histograms (obtained using the data of all layers) with specific characteristics pointing at different defect types. Different y-axis scales were chosen for improved readability. The micrographs show representative detail images parallel to the BD of each defect type.

The approach described in Section 2.2 was used to determine histograms of AlSi10Mg samples (cf. Section 2.1) using the data of all layers. Since different s were used, the total number of considered layers varies (331 layers for s=30 µm, 220 layers for s=45 µm, 166 layers for s=60 µm, and 109 layers for s=90 µm). The obtained histograms show different shapes and characteristics. These shapes and characteristics were found at all considered s, and can be related to different defect types as shown in Figure 3. At this point, it can be noted that samples processed with the same parameter combination show similar histograms.

**Figure 4:**
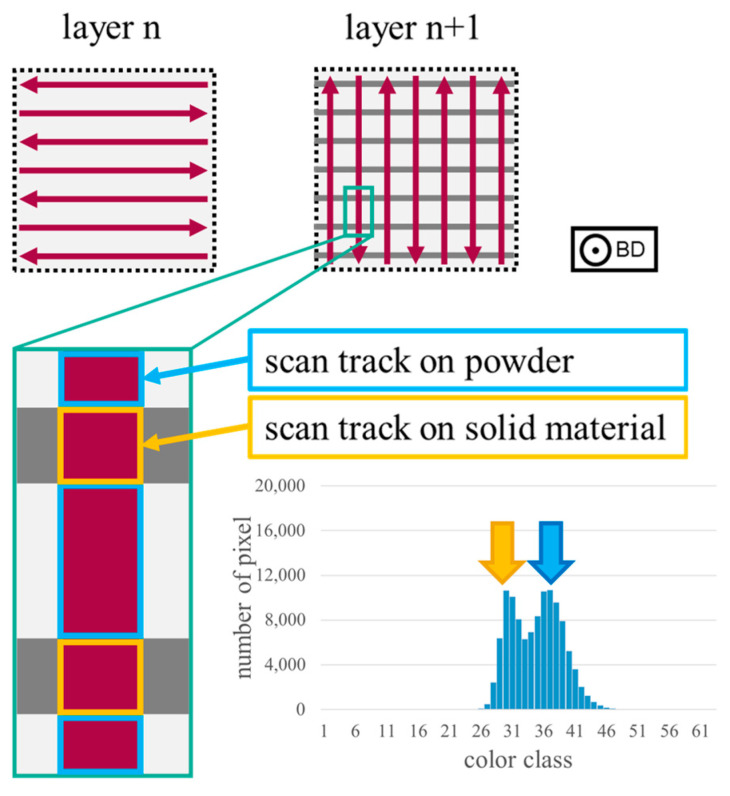
Schematic explanation for the formation of stripe pores and their influence on the corresponding histogram shape. Scan tracks on powder lead to values of a higher color class compared to scan tracks located on solid material. Since the distance between two scan tracks and the width of the scan tracks is in the same range, both cases result in a clear peak. Through the overlapping of those peaks, the double-peak in the histogram occurs.

Some histograms are characterized by two peaks, which are located close to each other and even have an overlap. The height of the peaks can be nearly the same or varying. This characteristic is referred to as double-peak in the following. A double-peak is often related to samples with stripe pores and, thus, to samples processed with a low E in combination with a wide h. In total, 42 of 44 (95.45%) samples with stripe pores show this histogram shape in the present study. A possible explanation for the double-peak is presented in Figure 4. Parallel to the BD, adjacent scan tracks form a lattice, which surrounds powder due to the applied scanning strategy and the wide h used. Thus, the laser exposure areas are located on powder and on solid material in each layer. Due to the different heat conductivities of powder and solid material, two different signals are measured. Based on the results of Alberts et al. [13], who stated that a scan track surrounded by powder leads to higher MPM values than a scan track surrounded by other scan tracks, the signal of areas being located on powder is expected to be higher than the signal of areas being located on solid material. Since the width of the scan tracks and the stripe pores is in a similar range, both signals appear in the histogram.

Other histograms are characterized by a single, narrow, high peak and can be related to almost fully dense material (porosity < 0.1%). Fully dense material could be achieved in an optimal E range that, however, depends on the used s. In total, 12 of 19 (63.16%) fully dense samples feature a histogram with such a narrow peak. The success rate in this case is lower since some histograms show a small peak indicating the evolution of keyholes (explained in the remainder of the text). Including these samples, the agreement increases to 84.21% (16 of 19 samples). The narrow peak pinpoints a constant signal measured by the MPM throughout the manufacturing sequence of the whole sample. This indicates stable processing conditions and, thus, a constant size and temperature of the melt pool. Eventually, this promotes low porosity and good SI [16]. These findings are supported by results presented in a study about the QM Meltpool 3D system [30]. In that study, it was reported that the distribution of the intensity signals obtained by the photodiode of the system for the whole sample is close to a normal distribution in case of a stable processing region [30]. The single, narrow, high peaks in the present study are indeed similar to a normal distribution.

Many histograms are characterized by a relatively flat main peak and an additional peak in color class 63, which summarizes the highest values measured. In the remainder of the text, this additional peak is referred to as 63-peak. The 63-peak is related to keyholes and high E, respectively. In total, 55 of 61 (90.16%) considered samples with keyholes have a histogram showing this peak. Presuming that a high MPM value can be related to a high temperature, the 63-peak is expected to indicate very high temperatures in the area of the melt pool and overheating, respectively [31]. Since the color scale was fixed to a specific value range in the beginning, the 63-peak does not mandatory indicate a saturation of the photodiode. Nevertheless, the 63-peak, as every other color class, summarizes measured signals with different values. As mentioned before, this represents a data reduction and details may be washed out. If raw data would be available, histograms with other, more detailed classes could be used and maybe further information could be drawn using this approach. Despite the data reduction by the used color classes, all measured values summarized in the 63-peak are very high and, therefore, indicate high temperatures and overheating.

Besides the main peak and the 63-peak being always present, some histograms obtained for samples with keyholes show a further small peak next to the main peak. This small peak always appears at lower color classes than the main peak, mainly in the range of color classes 23 to 33. It is observed for 38 of 61 (62.30%) samples with keyholes. The small peak could be related to the measured signals of the applied contour passes, which were lower than the signals from the remaining sample in these cases. Probably, the peak of the contour passes is present in all histograms, but is often overlaid by the signals of the bulk section of the samples.

Some histograms show combined characteristics, e.g., a double-peak in combination with a 63-peak (cf. row “combination of characteristics” in Figure 3). The corresponding samples are indeed characterized by mixed defect types. In the example mentioned, the sample suffered stripe pores due to a wide hatch as well as keyholes in the melt pools.

Although characteristics for different defect types could be identified, no specific characteristic could be assessed for samples with LoF so far. Histograms of corresponding samples are characterized by different characteristics like a double-peak or a 63-peak. However, the data base considered in the present work contains only a few samples with LoF. Further investigations with more samples showing this defect type could reveal a specific characteristic, since the results detailed above revealed that LoF defects do not lead to a “perfect” histogram shape (single, narrow, high peak). Moreover, LoF defects represent areas of unfused powder with a reduced heat conduction. Therefore, they should result in a heat accumulation in the following layer that should have an influence on the thermal signal, as detailed in [15]. However, Taherkhani et al. [19] could only detect LoF larger than 120 µm using the light intensity measured by EOSTATE MeltPool [19]. Accordingly, the detection of LoF is maybe limited by the specific characteristics of the defect itself.

### 3.2. Verification of the Presented Approach

**Figure 5:**
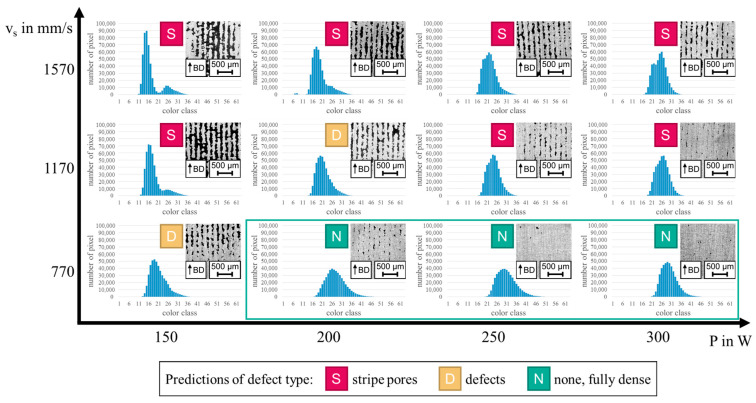
Histograms obtained from a full-factorial parameter study for PBF-LB/M-processed AlSi10Mg. The histograms were analyzed with respect to the characteristics described in Section 3.1 and predictions of the occurring defect types were made. Based on the predictions, samples with fully dense material could be identified (highlighted in green). The predictions were compared with the micrographs, revealing an overall good agreement.

Based on the histogram characteristics described in Section 3.1, a prediction of the occurring defect types in samples becomes possible. In the following, an example for such predictions is given using samples of a full-factorial parameter study for PBF-LB/M-processed AlSi10Mg with the aim to identify samples with fully dense material. The parameter study includes 12 samples produced with s=30 µm, h=0.2 mm, and different combinations of P and $v_{s}$. The processing conditions, porosity assessment, and the analysis of MPM data were equal to the procedures detailed in Section 2. The resulting histograms are shown in Figure 5. Each histogram was analyzed with respect to the characteristics described in Section 3.1 and a prediction of the occurring defect type was made without knowledge of the corresponding micrographs. In the following, some of those predictions are explained in more detail. As can be deduced from Figure 5, the histogram of the sample processed with P=150 W and $v_{s}=1570$ mm/s is characterized by a double-peak. The overlap of both peaks is small, nevertheless, the double-peak indicates stripe pores in the sample according to the results presented in Section 3.1. In contrast, the histogram of the sample processed with P=300 W and $v_{s}=1570$ mm/s shows a double-peak with a bigger overlap. This is similar to the double-peak shown in Figure 3 and indicates stripe pores, as well. Some samples have a histogram with a single peak. These histograms can be divided in two groups. Samples of the first group, e.g., the sample processed with P=150 W and $v_{s}=770$ mm/s, show an unsymmetrical peak. Eventually, these unsymmetrical peaks represent a kind of double-peak with a wide overlap, but they are definitely a sign for the presence of defects (not fully dense material). The second group of histograms with a single peak show a relatively flat, but symmetrical peak, e.g., the sample processed with P=300 W and $v_{s}=770$ mm/s. Although these peaks are not narrow and high, the shape that is reminiscent of a normal distribution and the absence of characteristics like a double-peak indicate fully dense material. Based on these predictions, process parameter combinations leading to fully dense material could be identified (highlighted in green). As can be seen by the micrographs presented in Figure 5, these predictions agree with the actual defects in most cases. Only one prediction was wrong, leading to an accuracy of 91.67%. This wrong prediction was made for the sample processed with P=200 W and $v_{s}=770$ mm/s. Here, the single peak in the corresponding histogram indicates fully dense material, but the micrograph reveals the presence of stripe pores. However, since the stripe pores are relatively narrow, the resulting porosity (0.79%) is low compared to other samples revealing this defect type.

Eventually, this example shows that the presented approach enables the prediction of occurring defect types in samples of parameter studies for the PBF-LB/M of AlSi10Mg with a high accuracy and without the use of conventional downstream testing methods. Based on that, the number of promising parameter combinations can be restricted so that only a few samples must be investigated in detail. In case of a build job with constant process parameters, e.g., for the manufacturing of similar parts, a rapid detection of components with defects is thought to be possible, since a specific parameter combination always leads to a similar histogram shape. Occurring defects eventually change this characteristic appearance. For example, powder bed inhomogeneities lead to local changes of s and, without an adaption of the process parameters, to defects [14]. Thus, a separation of parts with good quality from defective components is enabled so that a fast and non-destructive quality inspection becomes possible.

### 3.3. Influences on the Histograms

**Figure 6:**
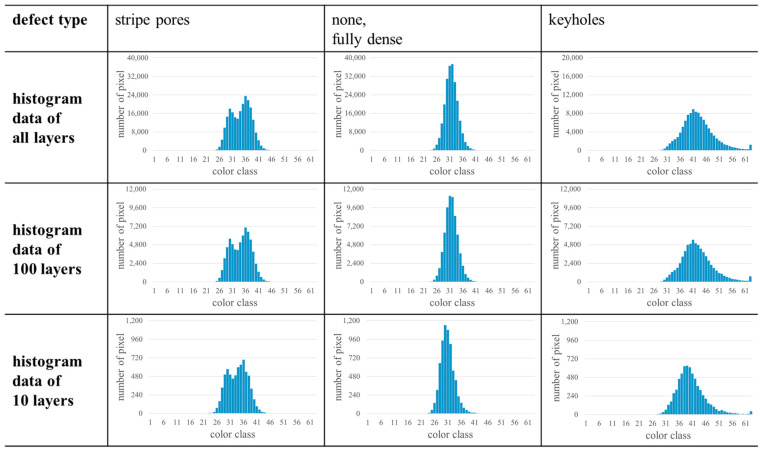
Representative examples of the evolution of histograms with increasing number of layers for different defect types. In case of stripe pores and fully dense material, 331 layers were considered for “all layers” (s=30 µm) and 166 layers in case of keyholes (s=60 µm). Different y-axis scales were chosen for improved readability.

**Figure 7:**
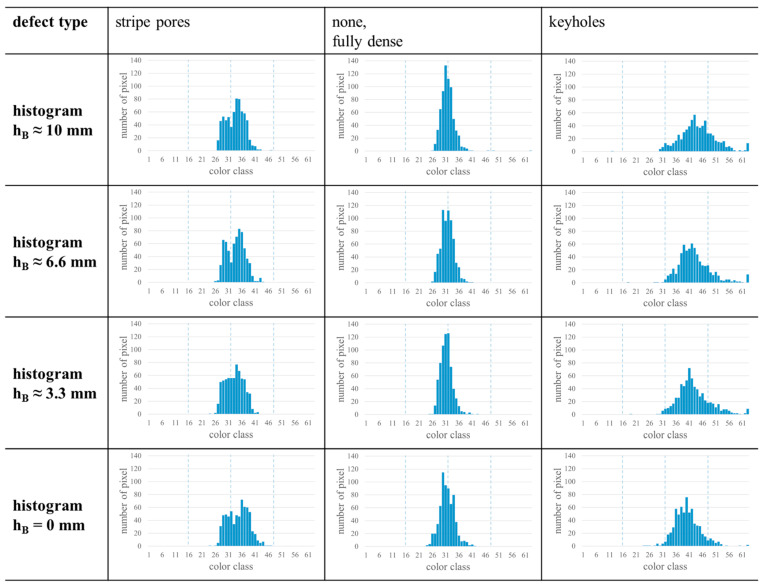
Histograms of single layers for different defect types at different build height $h_{B}$. The chosen samples are identical to the samples used in Figure 6.

In the investigations shown so far, all layers of the analyzed samples were considered. Since four different s were used, the total number of layers varies (cf. Section 3.1). Nevertheless, the characteristics in the histograms were observed for all s, so that the characteristics seem to occur independently of the number of considered layers. This would enable the use of the approach in combination with a low number of layers so that the approach would become even faster and more efficient. As a first step towards this, the histograms of a lower number of layers or even single layers are investigated in the following. Histograms determined based on a different total number of layers are shown in Figure 6, and histograms of single layers are summarized in Figure 7. As can be seen in Figure 6, the characteristics of the histograms occur even if only a small number of layers is analyzed. For example, a small 63-peak could be identified considering only 10 layers (cf. row “keyholes” in Figure 6). Nevertheless, the shape of the histograms turns out more clearly with an increasing number of layers. In principle, this is similar to e.g., X-ray measurements where peaks are more distinct with an increasing measurement time due to a better signal to noise ratio [32]. As a result, this preliminary assessment already indicates that the use of a small number of layers could be a promising way to further increase the efficiency of the presented approach. Nevertheless, further investigations have to be considered to identify the minimum number of layers leading to a reliable histogram for different materials.

Based on these results, a more detailed investigation of samples and parts becomes possible in practice, since information about the occurring defect type could be obtained from single layers, e.g., layers from a part section with very specific requirements. Moreover, a comparison of histograms from varying positions related to the build height $h_{B}$ might enable statements about changes in the defect development within the sample or, in the future, the variation of other properties with increasing $h_{B}$, e.g., porosity, residual stress, or hardness. To gain first insights into the variation of histograms with $h_{B}$, the results of single layers from different $h_{B}$ are shown in Figure 7. The characteristics in the histograms could be observed for different defect types. However, due to more pronounced noise, small deviations between the histograms of one sample occur. For samples with stripe pores, no further significant differences could be found between the histograms of different layers in the majority of the considered samples. Some fully dense samples and samples with keyholes as well have no significant differences in the histograms in case of different $h_{B}$ (cf. column “none, fully dense” in Figure 7). However, for some samples with these defect types, another behavior could be observed (cf. column “keyholes” in Figure 7). The histograms of those samples are shifting to higher color classes with increasing $h_{B}$. Following the considerations related to the MPM data mentioned earlier, this shift indicates that the temperature of the layers increases with increasing $h_{B}$. This is in good agreement with the effect of heat accumulation over $h_{B}$, which was observed in other studies, e.g., [33,34]. In addition to the shift of the histogram, the 63-peak becomes more pronounced with increasing $h_{B}$ in case of the example shown. Although this could be a side effect of the shifting histogram, it could be related to heat accumulation as well, since more keyholes would form as a result of overheating and the peak would become higher. However, this effect was rarely found among the probed samples. Moreover, some of the samples with keyholes show no 63-peak in the histograms of the considered single layers, although they have a 63-peak in the histogram composed of all layers. Eventually, this observation indicates that there are layers without keyholes. Future investigations will be conducted to confirm this thesis, so that layers with defects can be detected easily at the end.

**Figure 8:**
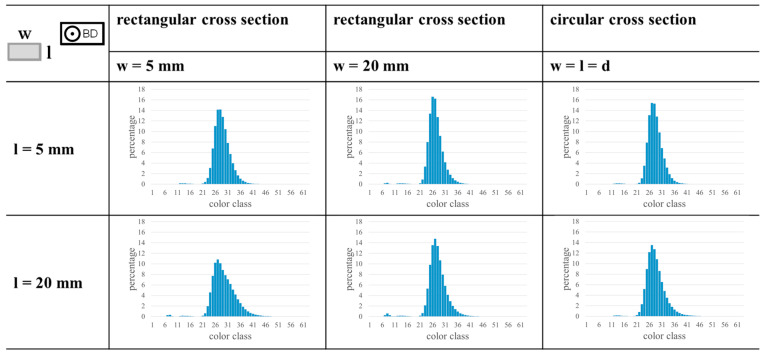
Histograms of samples with different cross sections for a variation of sample length l and width w, or the sample diameter d, respectively. A schematic shows the definition of l and w with respect to the build plate. The samples have a height of 10 mm and were produced with s=45 µm and process parameters leading to low porosity. The histograms were calculated using all layers of the build job.

Generally, the samples that were considered in the present study have relatively simple cross sections in form of squares. Accordingly, they do not adequately represent real parts with complex cross sections. Therefore, further investigations have to be made using more complex cross sections. As first step towards this, the approach presented was applied to data of AlSi10Mg samples that have different cross sections, i.e., a square, a rectangle, and a circle. The samples were processed using a parameter combination leading to low porosity (s=45 µm, P=299 W, $v_{s}=1267$ mm/s, and h=10 mm, cf. data publication of the previous study [25]). The processing conditions, porosity assessment, and the analysis of MPM data followed the procedure described in Section 2. All probed samples are characterized by a low porosity with a maximum of 0.24% for the largest cylindrical sample with a diameter of 20 mm. Using the approach introduced in the present study, the sample geometry could be identified correctly, and histograms could be determined for all samples as shown in Figure 8 (all layers of the samples were used to determine the histograms depicted). For comparability, the histograms were scaled using the total number of pixels of each sample. As a result, the histograms highlight the actual percentage value of each color class. All histograms have a single, narrow, high peak indicating fully dense material. This is in good agreement with the downstream measured porosity. The histograms of different samples vary slightly, e.g., the histogram of the sample with a length of 20 mm and a width of 5 mm shows a flatter and wider peak. The porosity of this sample (0.09%) is higher than the porosity of the corresponding sample with a length of 5 mm and a width of 20 mm (0.01%), eventually pointing at the influence of the sample orientation in the build chamber.

These results reveal that the approach is applicable for samples with different cross sections. However, it has to be noted that the considered cross sections are still relatively simple. Therefore, further investigations have to be made using even more complex cross sections and part geometries in the future. Especially, features that could influence the heat conduction situation, e.g., thin structures, up-skin areas, or down-skin areas, should be considered because changes in the heat conduction situation could also influence the measured MPM values [13]. Furthermore, in addition to the influence of complex geometries, another challenge concerning the application of the approach to real parts is seen. Different process parameters used in the fabrication of one layer are often applied. Different parameters are used for, e.g., up-skin areas, down-skin areas, or contour passes. As mentioned before, a small peak occurring in histograms of samples with keyholes could be linked to the applied contour passes. This indicates that each single parameter used in the process results in an own characteristic MPM signature. The individual histogram shapes overlap and form the resulting histogram of the complete sample. Based on this observation, it seems to be feasible to assign peaks to specific areas of process parameters, similar to the workflow in energy-dispersive X-ray spectroscopy. Samples with geometries requiring up-skin or down-skin areas, microstructurally graded samples, or samples only consisting of contour passes could be used to investigate this aspect in more detail in future.

### 3.4. Quantitative Statements

The results presented in the previous sections are based on the optical interpretation of the histograms. Although these interpretations can be used to determine defect types in samples with unknown properties (cf. Section 3.2), quantitative characteristics would be helpful for further development of the approach and further investigations. Therefore, first attempts focusing on various quantitative characteristics were made. Since other studies detailed correlations of the arithmetic mean and process parameters [13,21], which have a major influence on the resulting defect types and the porosity [24], a possible correlation of the arithmetic mean of the color classes in one sample and the occurring defect type was investigated. Although samples with stripe pores tend to have lower arithmetic means and samples with keyholes tend to have higher arithmetic means, the majority of the values are in a similar range so that a separation based on the arithmetic mean is not possible. Similar to these findings, no correlation of the arithmetic mean and the porosity could be found in the present study. This observation underlines that it is important to consider the histogram with its individual, specific characteristics. Therefore, quantitative values of the histograms were derived in the present work. A simple quantitative value of the histograms is the height of the peaks, e.g., the height of the narrow single peak or the height of the 63-peak (cf. Section 3.1). These values were assumed to correlate with the resulting porosity, e.g., the height of the 63-peak might indicate the ratio of keyholes and, thus, the degree of porosity. In some cases, there are signs that the peak height indeed provides information about the resulting porosity value, e.g., a higher narrow, single peak indicating lower porosity or a higher 63-peak indicating higher porosity. However, a clear correlation of the peak height and the resulting porosity could not be identified for the data assessed. In histograms with a double-peak, the ratio of the two peaks was assumed to correlate with the porosity since the peaks are probably caused by areas being located on powder and areas being located on solid material (cf. Figure 4). Accordingly, the height of the corresponding peaks might indicate the percentage of stripe pores or solid material in the sample, respectively. However, no correlation between the peak height ratio and the porosity could be found so far. These findings indicate that the reduction of the measured values represented by color classes to statistical values like the arithmetic mean is not sufficient to enable a prediction of the resulting defect type or to obtain correlations with the absolute degree of porosity. Eventually, indices that are characteristic for the histograms, like the entropy, could be promising candidates for quantitative characteristics. The entropy

(2) $$H=-\sum_{i=1}^{n}p_{i}\ln p_{i}$$

is calculated using the probability $p_{i}$ of n elements, assuming $\ln p_{i}=0$ for $p_{i}=0$. Related to the histograms of the present study, n=63 color classes were considered with $p_{i}$ being the percentage of color class i. The entropy was used in other studies, e.g., as representative feature of the distribution of intensity values for one layer measured with the photodiode of a QM Meltpool 3D system [35]. Although the entropy considers the percentage of each color class and, thus, in a certain way the shape of the histogram, a correlation to the occurring defect type or the porosity was not found in the present study.

These first attempts reveal that a single value seems to be not sufficient to describe the resulting defect types or porosity values based on the data obtained with the presented approach. The characteristics that can be easily detected by a human expert (cf. Section 3.1) seem to play an important role so that the histograms must be described as a whole, e.g., with a tool based on curve fitting. Although this is beyond the present study, it is an important part of future work since quantitative characteristics would be helpful for further development of the approach, as mentioned before. With the use of quantitative characteristics, an automated analysis of the histograms would become possible. This would be the first step toward a closed-loop implementation of the approach in the used PBF/LB-M system. The histograms of single layers could be analyzed automatically during the process and parts with identified failed layers could be removed from the build job. With access to the system, even a repairing step could be initiated. With further insights into the MPM data, similar approaches are conceivable to correct deviating residual stress states or mechanical properties.

Another approach to achieve quantitative statements concerning the resulting defect type or porosity could be the use of ML approaches. These approaches could be used to describe the histograms or to link the histograms with the resulting defect type or porosity, e.g., by using the number of pixels in each color class as input and the resulting porosity value as output. Moreover, ML approaches could enable a local analysis of the values saved in the 3D array, e.g., to locally detect porosity in the produced samples. Such a local detection of porosity is the aim of different studies, e.g., Taherkhani et al. [36] used the melt pool light intensity measured with the photodiode of an EOSTATE MeltPool system to detect porosity caused by the LoF phenomenon [36]. However, it must be considered that the data in the 3D array obtained by the presented approach are reduced by different reduction steps (cf. Section 2.2), so that the local analysis is limited to a certain resolution.

### 3.5. Transfer of the Presented Approach

**Figure 9:**
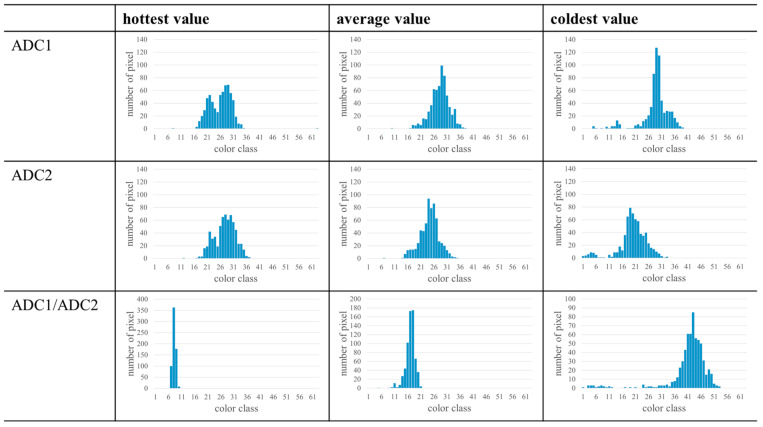
Histograms of a sample with stripe pores (s=30 µm) based on the different representations selectable in the MPM (cf. Figure 1). A layer from $h_{B}\approx 5$ mm was used to determine the histograms. The underlying screenshots were made with an individual color scale for each representation, which was adjusted automatically by the MPM.

The results presented are restricted to the measured values of the first photodiode (ADC1) of the MPM. Therefore, it is important to investigate the transfer to other photodiodes and other melt pool monitoring systems. Since two photodiodes are part of the MPM, the second photodiode (ADC2) could be used to investigate a possible transfer of the approach to another photodiode. Using a single layer at $h_{B}\approx 5$ mm from samples with s=30 µm (cf. Figure 1), histograms were determined for the values measured by the second photodiode (ADC2). The results of one sample with stripe pores are summarized in Figure 9. In principle, the histograms of ADC1 and ADC2 are characterized by a similar appearance, despite the more pronounced noise attributed to the fact that only a single layer is considered here. However, the histograms of ADC2 are located at lower color classes compared to the histograms of ADC1 for the “average value” mode and the “coldest value” mode. This could be related to the different photosensitivity of the photodiodes. Independent of the used photodiode, a double-peak could be identified in the “hottest value” mode. In contrast, different histogram shapes were observed in the other modes. The meaning of those shapes and their characteristics has to be investigated in future work. Nevertheless, the results indicate that the approach could be transferred to other photodiodes as long as a data reduction similar to the steps described in Section 2.2 is carried out.

Since the mounting and the function principle of the MPM are similar to a two-color pyrometer, and the measured ratio of such a pyrometer is a function of temperature independent of emissivity [37], the use of the ratios determined by the MPM could be a promising approach to gain further insights into the process and the measured data. Therefore, histograms of the ratio ADC1/ADC2 are shown in Figure 9. The histograms of ADC1/ADC2 in the “hottest mode” only consist of a few color classes. This corresponds to the uniform signal displayed in the MPM software (cf. Figure 1). Since all samples show nearly identical histograms for this mode, no distinction of defect types can be drawn. In contrast, the “coldest value” mode displays wider histograms with more details varying between the different samples. Eventually, characteristics could be identified for different defect types from this mode in future studies. Histograms of ADC2/ADC1 are not considered, since the (mostly) uniform signal of all samples would lead to (nearly) identical histograms so that no characteristics will be found for different defect types.

**Figure 10:**
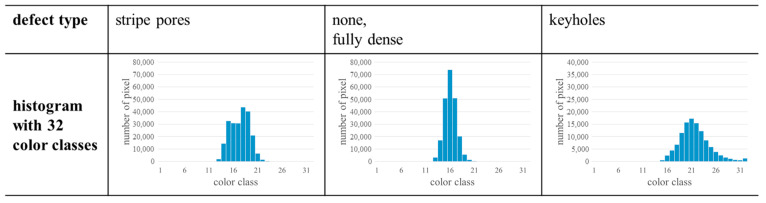
Histograms with 32 color classes. Based on the results shown before, the results of each color class were calculated by adding the number of pixels of adjacent color classes (color class 0 and color class 1, color class 2 and color class 3, etc.). The chosen samples are identical to the samples used in Figure 6 and Figure 7.

Since the detailed approach is a simplified way to obtain a distribution of the measured values, the approach could be transferred to other melt pool monitoring systems based on photodiodes. Often those other systems have a graphical representation of the data based on color scales. However, the color scales might use a different number of colors. In these cases, the approach could be used if it is adapted to the particular color scale. This could lead to a different number of color classes, however, other classifications, e.g., 32 classes, could also lead to similar results. To verify this, further investigations must be made in the future. As first step, the used MPM data could be shrunk from 63 to 32 color classes by adding the values of two adjacent color classes (examplary shown in Figure 10). Another way to obtain histograms could be the calculation based on raw data (if available). In this case, the raw data could be divided into 63 classes so that a histogram can be plotted. However, this must be investigated in the future explicitly focusing on the influence of the data reduction steps (cf. Section 2.2). Moreover, with access to the raw data, further assessments, e.g., based on ML could be implemented, and an additional local detection of defects could be enabled.

## 4. Summary and Conclusions

A novel approach was presented to analyze MPM data collected during the PBF-LB/M process. The approach uses layer-wise screenshots of the MPM software to determine the distribution of the measured values and to draw a histogram for each sample. Using this approach, data of the PBF-LB/M process of AlSi10Mg samples processed using different parameter combinations were analyzed. The resulting histograms could be related to the occurring defect types, and the identified characteristics can be used to predict the defect type in new and unknown samples. Different aspects of the presented approach were investigated. The results of the present study can be summarized as follows:

- The presented approach overcomes the obstacle of the missing export function in the MPM software and enables access to the data for every user. Additionally, the obtained data are reduced by data reduction steps included in the approach.
- Characteristics in the histograms of the obtained data could be related to the occurrence of defects and their types. A histogram characterized by a single, narrow, high peak is related to nearly fully dense material. Samples with defects show different histogram shapes, which are characterized by specific features for each defect type.
- Using the identified characteristics, a prediction of defect types in new and unknown samples becomes possible without the use of conventional downstream testing methods like metallography or µ-CT. Based on these findings, the presented approach enables a rapid and non-destructive quality inspection of PBF-LB/M-manufactured parts.
- The described characteristics of the histograms are already visible if a small number of layers is considered. On the one hand, this enables the prediction of defect types with a small number of layers and an increase in terms of the efficiency of the presented approach. On the other hand, more detailed investigations become possible, e.g., based on the assessment of histograms of single layers.
- Histograms of single layers can show individual features that may reveal information about the respective quality. Considering single layers, the histograms can change with increasing $h_{B}\approx 5$, eventually indicating processing issues, e.g., an increasing degree of overheating.
- The presented approach could be transferred to samples with more complex geometries, other photodiodes, or even other melt pool monitoring systems. However, these aspects must be investigated in detail in future studies.

## Data Availability

The raw data supporting the conclusions of this article will be made available by the authors on request.

## Supplementary Materials

The following supporting information can be downloaded at: https://www.mdpi.com/article/10.3390/ma17133287/s1. Table S1: Colors belonging to the color classes represented by the RGB values.
