# Supplementary material for: A Pragmatic Approach for Rapid, Non-Destructive Assessment of Defect Types in Laser Powder Bed Fusion Based on Melt Pool Monitoring Data
Source: Materials (Basel). 2024 Jul 3;17(13):3287. doi: 10.3390/ma17133287 (PMC11243436; doi:10.3390/ma17133287)
Supplement: Supplementary file 1 [file materials-17-03287-s001.zip › materials-3037984-supplementary.pdf]

Supplementary Material

A Pragmatic Approach for Rapid, Non-Destructive Assessment of Defect Types in Laser Powder Bed Fusion Based on Melt Pool Monitoring Data

A. Engelhardt[a], T. Wegener [a], T. Niendorf[a]

[a] Institute of Materials Engineering, Metallic Materials, University of Kassel, Mönchebergstr. 3, 34125 Kassel, Germany, E-mail: a.engelhardt@uni-kassel.de, t.wegener@uni-kassel.de, niendorf@uni-kassel.de

Corresponding author: A. Engelhardt, Mönchebergstr. 3, 34125 Kassel, Germany, E-mail: a.engelhardt@uni-kassel.de

Table S1 Colors belonging to the color classes represented by the RGB values.

	color class	R	G	B
	1	0	0	159
	2	0	0	175
	3	0	0	191
	4	0	0	207
	5	0	0	223
	6	0	0	239
	7	0	0	255
	8	0	15	255
	9	0	31	255
	10	0	47	255
	11	0	63	255
	12	0	79	255
	13	0	95	255
	14	0	111	255
	15	0	127	255
	16	0	143	255
	17	0	159	255
	18	0	175	255
	19	0	191	255
	20	0	207	255
	21	0	223	255
	22	0	239	255
	23	0	255	255
	24	15	255	239
	25	31	255	223
	26	47	255	207
	27	63	255	191
	28	79	255	175
	29	95	255	159
	30	111	255	143
	31	127	255	127
	32	143	255	111
	33	159	255	95
	34	175	255	79
	35	191	255	63
	36	207	255	47
	37	223	255	31
	38	239	255	15
	39	255	255	0
	40	255	239	0
	41	255	223	0
	42	255	207	0
	43	255	191	0
	44	255	175	0
	45	255	159	0
	46	255	143	0
	47	255	127	0
	48	255	111	0
	49	255	95	0
	50	255	79	0
	51	255	63	0
	52	255	47	0
	53	255	31	0
	54	255	15	0
	55	255	0	0
	56	239	0	0
	57	223	0	0
	58	207	0	0
	59	191	0	0
	60	175	0	0
	61	159	0	0
	62	143	0	0
	63	127	0	0
